# Automated deep learning-based arousal detection agrees with ten expert scorers in unseen data

**DOI:** 10.1093/sleep/zsag191

**Published:** 2026-07-16

**Authors:** Henna Pitkänen, Riku Huttunen, Masoumeh Tashakori, Marika Rissanen, Matias Rusanen, Heidur Gretarsdottir, Erna Sif Arnardottir, Tiina Paunio, Antti Kulkas, Sami Nikkonen, Timo Leppänen, Henri Korkalainen

**Affiliations:** Department of Technical Physics, University of Eastern Finland, Kuopio, Finland; Diagnostic Imaging Center, Kuopio University Hospital, Kuopio, Finland; Department of Psychiatry and SleepWell Research Program, Faculty of Medicine, University of Helsinki and Helsinki University Central Hospital, Helsinki, Finland; Department of Technical Physics, University of Eastern Finland, Kuopio, Finland; Diagnostic Imaging Center, Kuopio University Hospital, Kuopio, Finland; Department of Technical Physics, University of Eastern Finland, Kuopio, Finland; Diagnostic Imaging Center, Kuopio University Hospital, Kuopio, Finland; Clinical Research Center, Kuopio University Hospital, Kuopio, Finland; Department of Technical Physics, University of Eastern Finland, Kuopio, Finland; Diagnostic Imaging Center, Kuopio University Hospital, Kuopio, Finland; Department of Technical Physics, University of Eastern Finland, Kuopio, Finland; Diagnostic Imaging Center, Kuopio University Hospital, Kuopio, Finland; Clinical Research Center, Kuopio University Hospital, Kuopio, Finland; Reykjavik University Sleep Institute, School of Technology, Reykjavik University, Reykjavik, Iceland; Reykjavik University Sleep Institute, School of Technology, Reykjavik University, Reykjavik, Iceland; Department of Psychiatry and SleepWell Research Program, Faculty of Medicine, University of Helsinki and Helsinki University Central Hospital, Helsinki, Finland; Department of Technical Physics, University of Eastern Finland, Kuopio, Finland; Department of Clinical Neurophysiology, Seinäjoki Central Hospital, Seinäjoki, Finland; Department of Technical Physics, University of Eastern Finland, Kuopio, Finland; Diagnostic Imaging Center, Kuopio University Hospital, Kuopio, Finland; Department of Technical Physics, University of Eastern Finland, Kuopio, Finland; Clinical Research Center, Kuopio University Hospital, Kuopio, Finland; School of Electrical Engineering and Computer Science, The University of Queensland, Brisbane, Australia; Department of Technical Physics, University of Eastern Finland, Kuopio, Finland; Diagnostic Imaging Center, Kuopio University Hospital, Kuopio, Finland

**Keywords:** electroencephalogram, sleep fragmentation, arousal, interobserver agreement, automatic scoring, neural network, deep learning

## Abstract

**Study Objectives:**

Automatic arousal scoring is essential for the cost-effective analysis of polysomnograms. Many previous automatic methods have been tested in homogeneous, single-center datasets, and all against one scorer, on event-by-event basis, or in 30-s segments. We developed a deep learning-based arousal scoring model and tested it at 1-s resolution in an independent dataset scored by ten expert scorers from seven different sleep centers.

**Materials and Methods:**

A fully convolutional neural network model was developed using 1847 openly available polysomnograms from the Multi-Ethnic Study of Atherosclerosis (MESA) and tested in 205 hold-out polysomnograms from the MESA cohort, and in an independent Sleep Revolution (SR) cohort of 50 polysomnograms, each scored by ten scorers. Model performance was evaluated on an event-by-event basis and in 1-s segments. In the SR dataset, the performance was tested against each scorer individually and against their majority agreement.

**Results:**

In the MESA test set, the model achieved an event-by-event F1-score of 0.77, a 1-s segment *κ*-value of 0.67, and an arousal index (ArI) intraclass correlation coefficient (ICC) of 0.83. In the multi-scorer SR dataset, the model reached a median event-by-event F1-score, a 1-s *κ*-value, and an ArI ICC of 0.61, 0.50, and 0.66, respectively, exceeding the corresponding median values of the scorers among themselves (0.57, 0.46, and 0.59, respectively).

**Conclusions:**

The model achieved higher median arousal scoring agreement with ten manual scorers than the scorers did among themselves in an independent dataset. This suggests that the model generalizes well and can reliably be used in different sleep recordings.

## Graphical Abstract

**Graphical Abstract:**
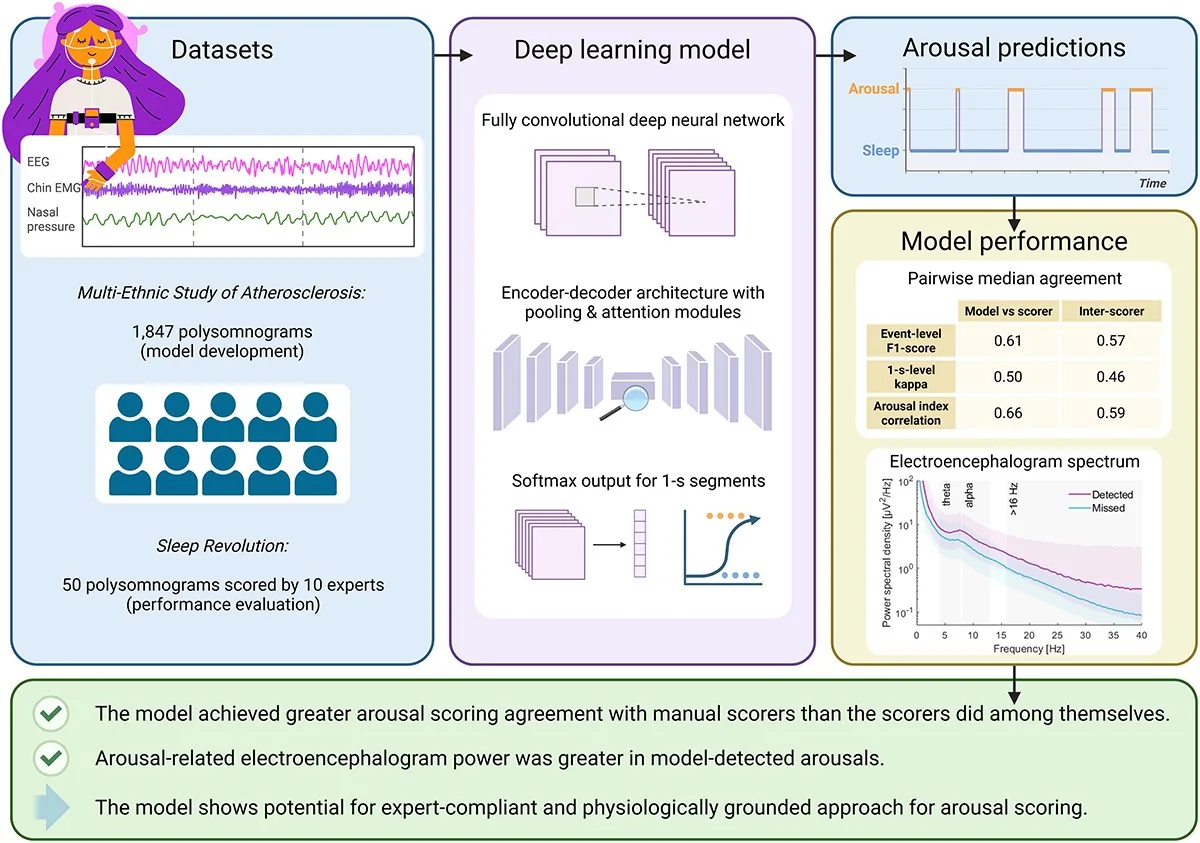

**Statement of Significance**

The presented automatic arousal detection model demonstrates potential toward clinical application for evaluating sleep fragmentation. Moreover, the presented model can reliably be used for research purposes to measure arousals in a consistent manner in large datasets and across different cohorts.

## Introduction

Arousals are brief wake-like episodes of neuroelectric activity that are both a part of healthy sleep and can in excessive amounts lead to sleep fragmentation and daytime sleepiness [1]. In the current clinical practice, arousals are identified from polysomnograms (PSGs) mostly by visual inspection based on the American Academy of Sleep Medicine (AASM) scoring rules [2]. The AASM defines an arousal as an abrupt ≥3-s shift in electroencephalogram (EEG) frequency including theta (4–7.99 Hz), alpha (8–13 Hz), and/or frequencies >16 Hz, and, in rapid eye movement (stage R) sleep, accompanied by an increase in the chin electromyogram (EMG) tone [2]. Interrater agreement in manual arousal scoring is relatively poor [3–5] compared to the agreement in the manual scoring of sleep stages [3, 6] and respiratory events [3, 7]. As arousals are embedded in the scoring rules of sleep stages, apneas, hypopneas, and periodic limb movements (PLMs) [2], uncertainties in arousal scoring can even affect the diagnostic accuracy of various sleep disorders, such as obstructive sleep apnea and PLM disorder.

Several automatic methods have been developed for arousal scoring using feature extraction with threshold-based decision criteria and/or traditional machine learning, as well as deep learning [8]. Deep learning-based methods for scoring AASM-defined arousals have been developed both with [9–12], and without EEG, for example only utilizing the electrocardiogram (ECG) [13] or respiratory inductance plethysmogram (RIP) [14]. In addition, several deep learning-based arousal scoring methods [8] have resulted from the PhysioNet 2018 Challenge [15]. However, most (99.6 per cent) of the target arousals in the Challenge were respiratory effort-related arousals (RERAs) [15]. As the rules regarding RERA scoring are distinct from the arousal rules [2], the models complying with the Challenge guidelines cannot be reliably compared against those aimed strictly for scoring arousals.

Previous studies have reported F1-scores of 0.70–0.81 for arousal detection in non-independent datasets (i.e. when the train and test sets came from the same PSG cohorts) [9, 11, 12] and 0.57–0.76 in independent datasets [10, 12, 14]. However, a limitation of most of the previous publications is that their performances were reported only on an event-by-event basis [9–13] or only using 30-s resolution [14], apart from one study that additionally reported a second-by-second F1-score of 0.68 in a non-independent dataset [9]. Moreover, in some studies, the model-detected arousals have been evaluated against manual arousal labels that have been prolonged from the original scorings [12]. Although the arousal index (ArI) can be computed based on the previous models, it is uncertain if the models are suitable for research purposes where precise arousal onset and offset scoring are required. Another limitation is that the performances of the previous models were evaluated against one scorer per recording. Additional testing in independent PSGs recorded in different centers using various equipment and scored by different scorers would evaluate the generalizability of the model more reliably. Moreover, testing in recordings scored by multiple scorers would effectively evaluate the model against a probability distribution of manually scored arousals, reflecting how the model compares to uncertainties in the manual scoring.

The objective of this study was to develop a deep learning model for the automatic detection of arousals using EEG, chin EMG, and nasal pressure signals, and to evaluate the model in independent data of 50 PSGs, each annotated by 10 different expert scorers from seven different sleep centers across Europe and Australia. This allows extensive evaluation of the model against a distribution of manual scorers instead of relying on a single reference label. The aim was to evaluate the model’s performance with no other label annotation post-processing than implementing the AASM arousal scoring rules (minimum duration of 3 s with 10 s of stable sleep prior to onset). We aimed to evaluate the performance both on an event-by-event basis and in more detailed 1-s resolution, using the original individual scorer annotations as well as subsets of arousals with high or low agreement among multiple scorers. An additional aim was to compare the EEG spectral power in the arousal-related alpha, theta, and >16 Hz frequencies [2] between arousals scored and missed by the automatic model. The first hypothesis was that the model can score arousals similarly to the manual scorers and that its performance is on par with the manual scorings. The second hypothesis was that the EEG alpha, theta, and >16 Hz frequencies are more pronounced in arousals scored by the model than in those not scored by the model.

## Materials and Methods

An overview of the methodological workflow of this study is presented in Figure 1.

**Figure 1:**
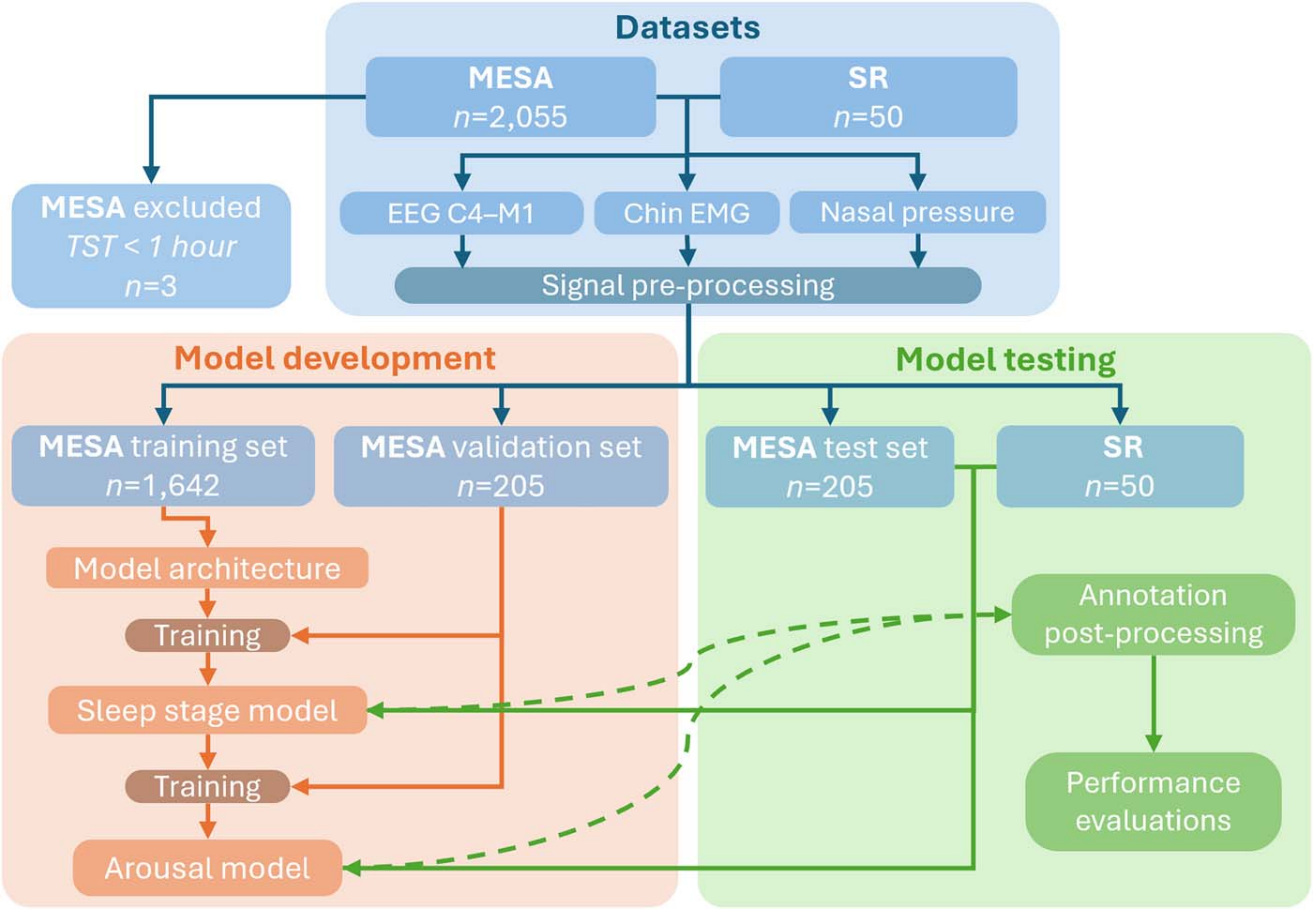
The methodological data- and workflow of the study. The Multi-Ethnic Study of Atherosclerosis dataset was divided into separate training, validation, and test sets in such way that they did not comprise same subjects. The Sleep Revolution dataset comprised 50 polysomnograms, each scored by 10 individual scorers. EEG = electroencephalogram, EMG = electromyogram, TST = total sleep time.

### Datasets

#### Multi-ethnic study of atherosclerosis dataset

The model was developed by utilizing 2055 type II PSGs from the Multi-Ethnic Study of Atherosclerosis (MESA), collected in various institutions in the United States between 2010 and 2013 [16–18]. The PSGs were recorded with the Compumedics Somté system (Compumedics Ltd, Abbotsford, Australia) [17], and they included EEG, electrooculogram (EOG), chin EMG, ECG, peripheral pulse oximetry, thoracic and abdominal RIP, thermocouple, nasal pressure, leg movement, and body position signals. The data collection was approved by the review boards of all participating institutions, and all subjects gave informed written consent [17]. The PSGs were scored based on the AASM 2007 [19] scoring rules and the PSG data and annotations were provided in the European Data Format (EDF). After excluding three subjects with ˂1 h of total sleep time (TST), the MESA dataset (*n* = 2052) was divided randomly into separate training (*n* = 1642), validation (*n* = 205), and test (*n* = 205) sets. Demographic information on the MESA dataset is presented in Table 1, and more information is provided by the National Sleep Research Resource [18].

**Table 1:** Demographic information of the population in the Multi-Ethnic Study of Atherosclerosis dataset (*n* = 2052)

	Count (%)			
	*Whole population*	*Training set*	*Validation set*	*Test set*
Male	952 (46.4)	758 (46.2)	100 (48.8)	94 (45.9)
Female	1100 (53.6)	884 (53.8)	105 (51.2)	111 (54.1)
	**Median (IQR)**			
	***Whole population***	***Training set***	***Validation set***	***Test set***
Age [years]	68.0 (62.0–76.0)	68.0 (62.0–76.0)	68.0 (62.0–76.3)	68.0 (61.0–76.0)
BMI [kg/m^2^]	27.9 (24.6–31.9)	27.9 (24.7–31.9)	27.7 (24.4–31.5)	28.1 (24.5–32.1)
AHI [1/h]	32.8 (20.3–50.7)	32.9 (20.4–50.6)	29.6 (18.5–50.1)	35.4 (20.3–53.4)
ArI [1/h]	24.0 (17.1–33.8)	24.0 (17.3–33.8)	22.5 (16.5–31.8)	25.2 (16.5–34.1)
TST [h]	6.1 (5.2–6.9)	6.2 (5.3–6.9)	6.1 (5.1–6.9)	6.1 (4.9–6.8)
N1 [% of TST]	12.0 (8.2–17.8)	11.9 (8.4–17.6)	12.4 (7.6–18.7)	11.9 (8.0–18.0)
N2 [% of TST]	58.2 (51.5–64.5)	58.3 (51.7–64.5)	57.0 (49.4–63.9)	58.4 (52.2–64.8)
N3 [% of TST]	8.0 (1.9–15.4)	8.0 (1.9–15.3)	8.7 (1.9–16.4)	8.2 (2.0–15.6)
R [% of TST]	18.3 (13.6–22.3)	18.3 (13.7–22.4)	18.6 (13.1–23.1)	17.9 (13.9–20.8)

AHI = apnea-hypopnea index, ArI = arousal index, BMI = body mass index, IQR = interquartile range, N1 = non-rapid eye movement stage 1, N2 = non-rapid eye movement stage 2, N3 = non-rapid eye movement stage 3, R = rapid eye movement stage, TST = total sleep time.

#### Sleep Revolution dataset

The performance of the model was tested in an independent multi-scorer dataset, collected in Iceland during 2021 as part of the Sleep Revolution (SR) project [20]. The SR dataset comprised 50 type II PSGs, recorded with the Nox A1 device (Nox Medical, Reykjavik, Iceland). The recordings included EEG, EOG, chin and leg EMGs, ECG, peripheral pulse oximetry, thoracic and abdominal RIP, nasal pressure, and body position measurements. The collection of the SR data was approved by the National Bioethics Committee of Iceland (21-070, 16/3/2021), and all participants gave informed written consent. Each PSG was scored by ten experienced sleep specialists from seven sleep centers in Europe and Australia, using the AASM 2020 [21] guidelines and being blinded to each other’s scorings. The PSG data and annotations were exported as EDFs from the scoring software (Noxturnal Research version 6.1.0.30257, Nox Medical, Reykjavik, Iceland). Demographic information on the SR population is presented in Table 2. More detailed information on the subject demographics, scorer acquisitions, and the recording and scoring procedures are provided in previously published studies on the manual scoring agreement in the same dataset [5–7].

**Table 2:** Demographic information of the population in the Sleep Revolution dataset (*n* = 50)

	Count (%)
Male	29 (58.0)
Female	21 (42.0)
	**Median (IQR)**
Age [years]	42.5 (32.0–52.0)
BMI [kg/m^2^]	26.7 (23.6–31.0)
	**Median (IQR)** ^**a**^
AHI [1/h]	9.2 (4.0–19.0)
ArI [1/h]	17.1 (12.6–20.7)
TST [h]	7.1 (6.4–7.9)
N1 [% of TST]	7.1 (6.2–9.8)
N2 [% of TST]	48.2 (43.1–51.4)
N3 [% of TST]	22.7 (17.4–26.7)
R [% of TST]	20.8 (18.0–23.5)

^a^Computed as the median and IQR of the interrater medians of each subject.

AHI = apnea-hypopnea index, ArI = arousal index, BMI = body mass index, IQR = interquartile range, N1 = non-rapid eye movement stage 1, N2 = non-rapid eye movement stage 2, N3 = non-rapid eye movement stage 3, R = rapid eye movement stage, TST = total sleep time.

### Signal preprocessing

Both MESA and SR datasets were formatted from EDFs into the unified SleepLab Format (SLF) [22] for standardized annotation and signal handling, and easier integration. Three signals were chosen from both datasets as inputs to the model: EEG channel derivation C4–M1 (to capture arousal-related EEG frequency shifts), chin EMG (to capture arousals in REM state), and nasal pressure (a supporting signal for respiratory arousals) [2]. The sampling frequencies of the EEG and chin EMG signals were downsampled from the original 256 Hz (MESA) and 200 Hz (SR) to 64 Hz. Before downsampling, the EEGs were high- and lowpass filtered to 0.3–32 Hz, and the chin EMGs to 10–32 Hz, complying with the AASM guidelines [2]. Nasal pressure signals of the SR dataset were high- and lowpass filtered to 0.1–32 Hz and downsampled from the original 200 Hz to 64 Hz. Nasal pressure signals of the MESA dataset were only highpass filtered to 0.1 Hz, as they had already been lowpass filtered to 10 Hz in the hardware [18]. After filtering, they were upsampled from the original 32 Hz to 64 Hz sampling frequency. All signal filtering was executed using 5th-order Chebyshev type II digital filters and all signal resampling was executed using linear interpolation. Finally, z-score normalization was used for the whole-night signals to standardize them to a zero mean and a variance of one.

### Model architecture

The model was developed on an existing architecture initially presented by Huttunen et al. [23], comprising a fully convolutional encoder-decoder-structured deep neural network (based on the U-Time [24]). The architecture also included channelwise attention mechanisms (Squeeze-and-Excitation [25]) and feature extraction modules (Atrous Spatial Pyramid Pooling [26]). Previous models utilizing this architecture have demonstrated state-of-the-art performances in scoring sleep stages and respiratory events in multiple datasets [23, 27–29]. More detailed information on the model architecture can be found in [23, 27]. The present model architecture was otherwise similar to the initial study [23], but unlike the original model with two separate outputs (one for sleep staging and one for respiratory events), the present architecture was used to train two separate models with one output each (one for sleep stages and one for arousals).

### Model training

#### Sleep stage model training

First, the sleep staging model was trained using EEG, chin EMG, and nasal pressure signals from the MESA training set, with 1-s segment length. As the manually scored hypnograms comprised 30-s epochs, the labels of each epoch (wake “W”; non-rapid eye movement “N1,” “N2,” or “N3”; or rapid eye movement “R”) were replicated into 30 consecutive 1-s segment labels.

The model’s weights were adjusted during 100 training epochs in batches of 8 randomly selected recordings by choosing 2 h of consecutive signal data starting at a random time point in each recording. Similarly to the initial study [23], the weight adjustment was conducted using stochastic gradient descent with momentum and decoupled weight decay. For the sleep stage model, a stable learning rate of 0.01 was used during the first 80 epochs of training, and during the final 20 epochs the learning rate was exponentially decreased with a decay factor of 0.9. Like the initial publication [23], categorical cross-entropy was used as the loss function for the sleep stage model. The final sleep stage model was chosen based on the minimum loss in the MESA validation set, computed after each training epoch.

#### Arousal model training

The arousal scoring model was trained in the MESA training set using the same signals and segment length as the sleep stage model. The weights of the sleep stage model were used as the initial weights for the arousal model without freezing any layers. Only the last layer of five weights, responsible for the labeling of the five sleep stages, was replaced with a layer of two randomly initialized weights to distinguish only between two labels (“non-arousal” or “arousal”).

During the arousal model training, its weights were adjusted similarly to the sleep stage model, apart from the learning rate and loss function. The learning rate for the arousal model was linearly increased from 0.0001 to 0.001 during the first 40 epochs, then linearly decreased back to 0.0001 during the following 40 epochs, and finally exponentially decreased with a decay factor of 0.9 during the last 20 epochs. Due to class imbalances between arousal and non-arousal labels, as arousals only represent 1.1–4.2 per cent of PSGs [5], alpha-balanced focal cross-entropy [30] was used as the loss function when training the arousal model. To place more emphasis on the arousal than the non-arousal class, the weight factor, i.e. the alpha value, was set to 0.75 for arousals and 0.25 for non-arousals, and the focusing parameter, i.e. the gamma value, was set to 2. The final arousal model was chosen based on the minimum loss in the MESA validation set, computed after each training epoch.

### Annotation postprocessing

For the final performance evaluation in the MESA test set, the region of interest was restricted between the “lights out” and “lights on” times. In the SR dataset, the region of interest was restricted to periods where all ten manual scorers were actively scoring, i.e. from the last manual hypnogram start to the first manual hypnogram end.

The sleep staging model outputs were the softmax scores representing the sleep stage probabilities during each second of the recording, out of which the label with the highest softmax value was chosen as the predicted sleep stage. To attain comparable TSTs with the manual scorers, who had annotated their sleep stages in 30-s epochs, the automatically scored hypnograms were downsampled into 30-s epochs by combining 30 consecutive 1-s segments. The sleep stage for each 30-s epoch was chosen based on the most frequently occurring sleep stage in the 1-s segments.

The arousal model outputs were the softmax values representing the “arousal” and “non-arousal” probabilities during each second of the recording, out of which the label with the higher softmax value (i.e. model confidence) was chosen. As the arousal duration is at least 3 s [2], a segment was considered as an arousal if it comprised ≥3 consecutive arousal seconds. Moreover, as at least 10 s of uninterrupted sleep should be scored before an arousal [2], if there were <10 consecutive non-arousal seconds between two arousals, the latter arousal was discarded.

### Model performance evaluation

The performance of the sleep staging model was tested in both datasets; MESA and multi-scorer SR, to ensure sufficient reliability for using the automatically scored TSTs in further analyses. Accuracy, precision, recall, and F1-scores [31] were computed from the 30-s epochs labeled either wake (“W”) or sleep (combined “N1,” “N2,” “N3,” and “R”) between the automatic and manual hypnograms in the MESA dataset, and between the automatic and the majority hypnograms in the SR dataset. The majority hypnograms were formed as the most scored sleep stage by the manual scorers in each 30-s epoch, further explained in [6].

ArIs in the MESA test set were computed using the manual TSTs for both the manually and automatically scored ArIs. In the SR dataset, the TSTs for the automatically scored ArIs were determined by the sleep staging model of this study, and for the manual ArIs, the TSTs were determined by each scorer in question. ArI agreement was investigated using intraclass correlation coefficients (ICCs), calculated with a two-way mixed effects model for the absolute agreement between single measurements (ICC(A,1) in [32]) in both datasets, with additional mean absolute error (MAE) in MESA. Moreover, the arousal durations, start and end time differences of overlapping arousals, and the number of arousals starting in different majority sleep stages were compared between the automatic model and the individual manual scorers in the SR dataset.

In addition to the individual scorers, the performance in the SR dataset was evaluated against arousal sequences by multiple scorers. An arousal sequence started at the onset of the first coinciding arousal by any manual scorer and ended at the termination of the last coinciding arousal, if there were any, by any manual scorer. The level of agreement in each arousal sequence was evaluated by investigating the number of manual scorers scoring the arousals in the same sequences. The automatic model was considered to have identified a sequence if a detected arousal by the model overlapped with that sequence.

The 1-s performance of the arousal scoring model was tested both in MESA and multi-scorer SR datasets using confusion matrices, precision, recall, F1-scores, and Cohen’s *κ* [33] values. Precision, recall, and F1-scores were also computed on an event-by-event-basis, such that a predicted arousal was considered a true positive event if it overlapped with a manually scored arousal. In the SR dataset, the model performance was evaluated both against the individual scorers, and against the manual scorer majority. Manual majority for the 1-s performance was formed by labeling 1-s segments as “arousal” only if ≥5 manual scorers scored an arousal during that second. For the event-by-event performance, the majority arousal events were formed from the arousal sequences by ≥5 manual scorers. In addition to the majority arousal events, event-by-event F1-score was evaluated at different scorer-agreement levels, i.e. against arousal sequences formed by different numbers of scorers.

### E‌EG signal power in manually and automatically scored arousals

EEG signal power was investigated during the arousal sequences in the multi-scorer SR dataset. For these analyses, the original EEG sampling frequency (200 Hz) was used, and no filtering was applied. EEG power spectral density (PSD), i.e. EEG frequency distribution within unit time, of each arousal sequence was computed as


$$\mathrm{PSD}_k\left\{x \left(t\right)\right\}=\left\{\begin{array}{@{}c}\dfrac{{\left|\mathrm{FFT}_k\left\{x\left(t\right)\right\}\right|}^2}{f_sN},\kern1em k=1,N/2+1\\[3mm] {}\ \dfrac{2{\left|\mathrm{FF}{\mathrm{T}}_k\left\{x(t)\right\}\right|}^2}{f_sN},\kern1em k=2,\dots, N/2\end{array}\right.,$$

where $\mathrm{FF}{\mathrm{T}}_k\left\{x(t)\right\}$ is the fast Fourier transform [34] of the EEG segment *x(t)*, *t=1,…, N*, at a frequency bin *k*, and ${f}_s=200$ Hz is the sampling frequency. To obtain equal lengths for the PSDs, they were resampled with linear interpolation to $3{f}_s/2+1=301$ frequency bins, corresponding to a PSD of a 3-s EEG segment. PSD medians were computed separately for stages W, combined N1–N3, and R in groups based on (1) whether the automatic model scored the arousal or not and (2) whether either the majority (≥5) or minority (≤4, including zero when no manual scorers agreed with the model) of the manual scorers scored the EEG segment as an arousal.

EEG signal power was computed as the area under the PSD curve within frequency bands theta (4–7.99 Hz), alpha (8–13 Hz), and >16 Hz (16–40 Hz in the present analyses) using numerical integration via the trapezoidal method. Statistical differences in the band powers were investigated for cases (1) and (2), separately within each sleep stage (W, combined N1–N3, and R), with the Wilcoxon signed-rank test. Due to uneven group sizes, the median *p*-value of 5000 random pair permutations between the compared groups was computed. A threshold of *p* < .05 was set as the limit of statistical significance.

## Results

### Model performance

The event-by-event recall and precision of the automatic arousal scoring model in the MESA test set were 0.82 and 0.72, respectively (Table 3). The MAE between the automatically and manually scored ArIs in the MESA test set was 5.48 1/h, and the ArI ICC was 0.83. In the independent multi-scorer SR dataset, the event-by-event recall and precision against the majority arousal events were 0.80 and 0.69, respectively (Table 3). Furthermore, the second-by-second *κ* was 0.67 in the MESA test set and 0.60 in the SR dataset against the majority arousal seconds (Figure 2).

**Table 3:** Classification reports of the automatic arousal scoring and sleep staging models in the Multi-Ethnic Study of Atherosclerosis and the multi-scorer Sleep Revolution datasets

MESA: *205 polysomnograms, each scored by one out of three scorers*							
*Model*	*Classification*	*Class*	*Precision*	*Recall*	*F1*	*n (true)*	*n (predicted)*
arousal scoring	1-s segments	Non-arousal	0.98	0.98	0.98	6 004 588	5 957 350
		Arousal	0.65	0.73	0.69	371 042	418 280
	event-by-event^a^	Arousal	0.72	0.82	0.77	32 801	37 224
sleep staging	30-s segments	Sleep	0.98	0.91	0.94	144 821	134 490
		Wake	0.83	0.95	0.88	67 700	78 031
**SR: *50 polysomnograms, each scored by ten scorers*^b^**							
*Model*	*Classification*	*Class*	*Precision*	*Recall*	*F1*	*n (true)*	*n (predicted)*
arousal scoring	1-s segments	Non-arousal	0.99	0.97	0.98	1 332 963	1 306 261
		Arousal	0.50	0.79	0.62	47 012	73 714
	event-by-event^a^	Arousal	0.69	0.80	0.74	5111	5941
sleep staging	30-s segments	Sleep	0.98	0.96	0.97	41 535	40 505
		Wake	0.69	0.85	0.77	4464	5494

^a^Predicted event was considered as a true positive event if it overlapped with a manually scored event.

^b^Agreement was tested against the majority score, i.e. 5–10 manual scorers agreeing.

**Figure 2:**
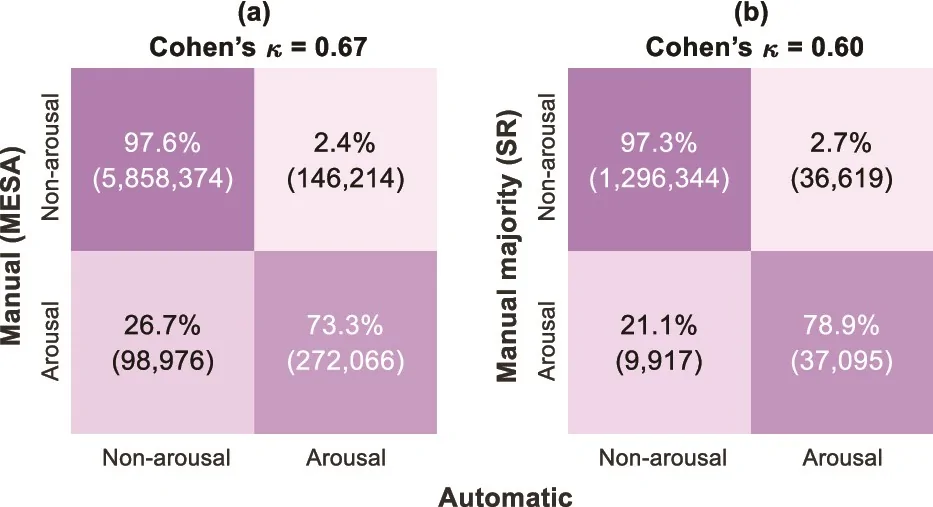
The confusion matrices and Cohen’s kappa (*κ*) values computed based on 1-s segments in the (a) Multi-Ethnic Study of Atherosclerosis (*n* = 205) and (b) multi-scorer Sleep Revolution (*n* = 50, with ten scorers each) test sets. In (b), the model performance was compared against the majority arousal seconds, i.e. at least five out of ten scorers agreeing during each 1-s segment.

For the automatic TST definition, the 30-s wake/sleep accuracy of the sleep staging model was 92.0 per cent in the MESA test set and 94.9 per cent in the SR dataset when compared against the majority sleep stages. The 30-s precision, recall, and F1-scores of the sleep staging model are reported in Table 3.

In the multi-scorer SR dataset, arousals scored by the automatic model and the manual scorers followed similar trends in ArI, arousal duration, and distribution across sleep stages (Figure 3). The median duration of the automatically scored arousals was 9.0 s compared to 6.9 s for manually scored arousals. Moreover, the median start and end time differences between the overlapping automatically and manually scored arousals were –1.3 s and +0.9 s, respectively, meaning that the automatically scored arousals typically started before and ended after their manually scored counterparts. Furthermore, the model scored arousals in different sleep stages similarly to the manual scorers. The second-by-second *κ*-values between the model and the majority arousal seconds were 0.52, 0.59, 0.60, 0.64, and 0.62 in the majority stages W, N1, N2, N3, and R, respectively.

**Figure 3:**
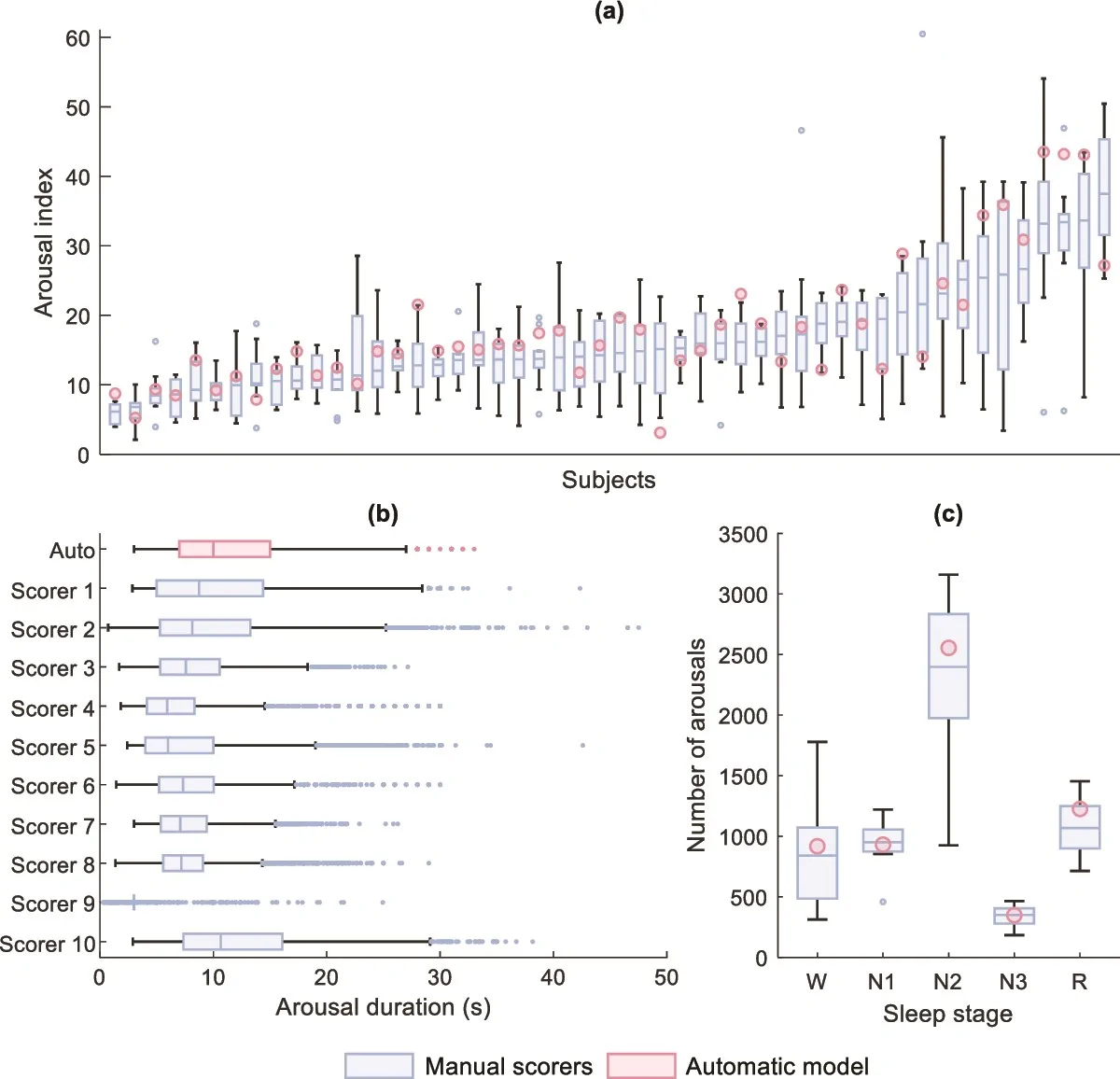
(a) Arousal indexes (ArIs), (b) arousal durations, and (c) the number of arousals in stages wake (W), non-rapid eye movement (N1, N2, and N3), and rapid eye movement (R), scored by 10 manual scorers and the automatic model in 50 subjects. In (a), the total sleep times (TSTs) for the manual ArIs were determined by each scorer in question, whereas the TSTs for the automatic ArIs were determined by the sleep staging model. Moreover, in (a), the subjects were sorted in ascending order according to the manual ArI medians. Sleep stages in (c) were determined based on the manual majority hypnograms for both manually and automatically scored arousals.

The median ICC between the model’s and the individual manual scorers’ ArIs was 0.66, and the median second-by-second *κ* was 0.50 (Figure 4), whereas the corresponding median values between the manual scorers themselves were 0.59 and 0.46, respectively. Moreover, the median event-by-event F1-score of the model was 0.61 against the individual manual scorers (Figure 5) and 0.74 against the majority arousal events (Table 3), whereas the corresponding median values of the manual scorers themselves were 0.57 and 0.73, respectively. Furthermore, the agreement level-wise event-by-event F1-scores of both the automatic model and the manual scorers peaked at four agreeing scorers, where true and predicted event counts were also the most similar (Figure 6). However, the model’s confidence increased monotonically with scorer agreement.

**Figure 4:**
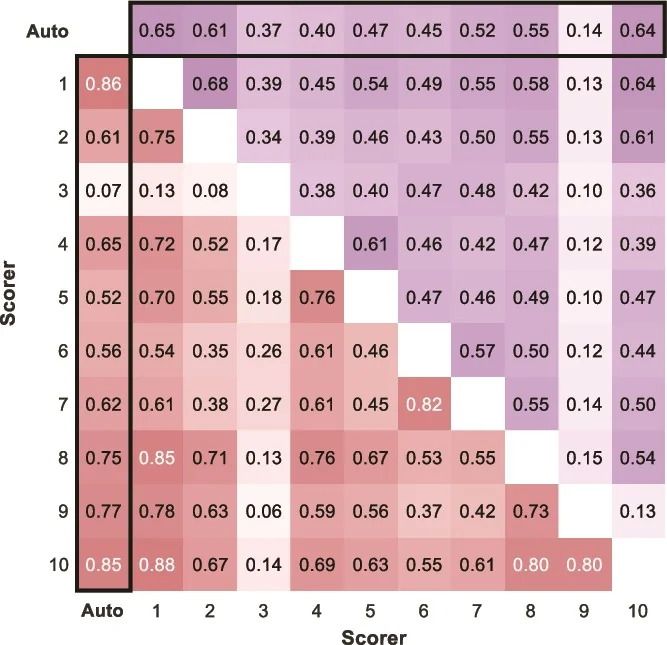
Pairwise intraclass correlation coefficients of arousal indexes (lower left) and Cohen’s kappa values of 1-s arousal/non-arousal segments (upper right) of ten scorers and the automatic model in 50 polysomnograms. Automatic model versus manual scorer -pairs are emphasized with rectangles.

**Figure 5:**
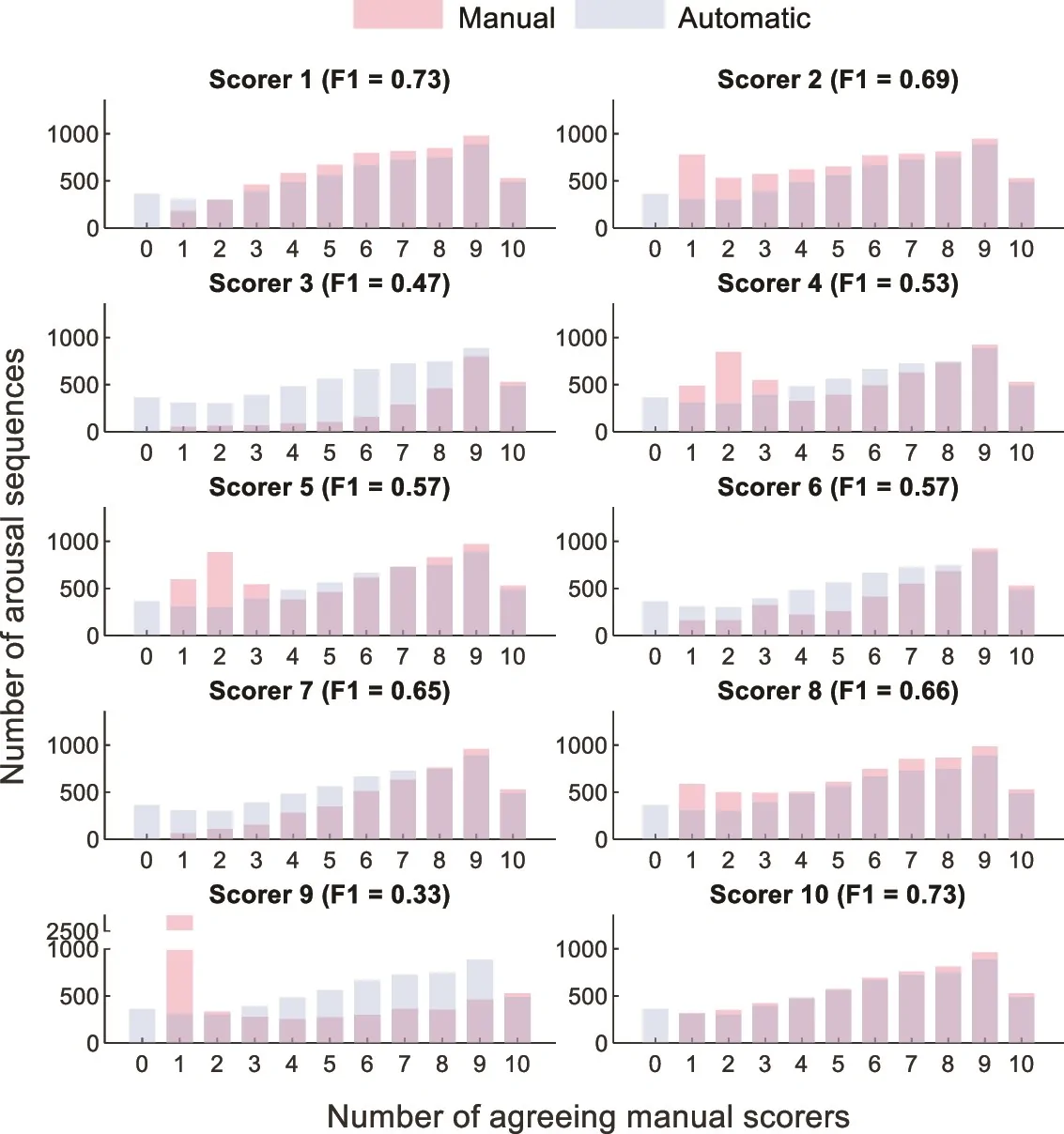
Arousals scored by the automatic model and the 10 manual scorers, grouped by the number of manual scorers scoring the arousal. The *y*-axis and the bar corresponding to 1-scorer agreement of scorer 9 have been truncated and erased between 1000 and 2500 sequences to match the *y*-axis scale of the rest of the scorers. Event-by-event F1-scores were calculated by treating an automatically scored arousal as a true positive if it overlapped with a manually scored one by each scorer in question.

**Figure 6:**
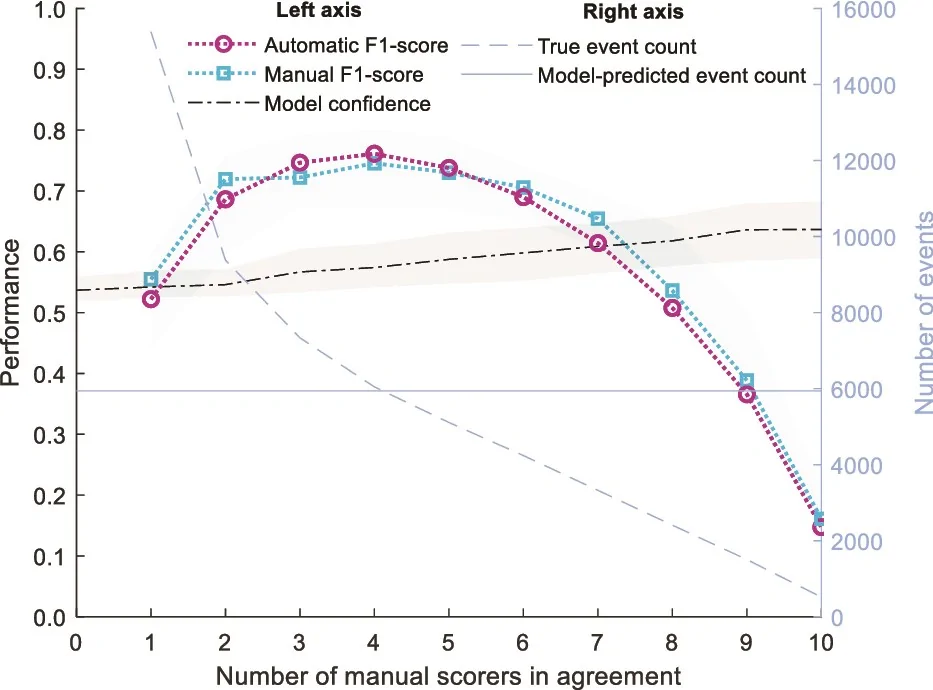
Event-by-event F1-scores of the automatic model and manual scorers (latter as median with shaded interquartile range), model confidence (as median with shaded interquartile range), and true and model-predicted event counts, as a function of manual scoring agreement.

A total of 15 304 arousal sequences, i.e. groups of coinciding arousals by one or more scorers, were formed in the multi-scorer SR dataset (Figure 5 and Table 4). Out of these sequences, the model detected 5402 and disregarded 9902. However, most (57.1 per cent) of the sequences the model disregarded were formed based on only 1 manual scorer scoring an arousal. A third (32.0 per cent) of the sequences the model disregarded had 2–4 manual scorers, and the minority (10.9 per cent) had 5–10 manual scorers scoring an arousal. On the other hand, most of the sequences the model detected (74.7 per cent) were also scored by 5–10 manual scorers. A fifth of the sequences the model detected (20.3 per cent) included arousals by 2–4 manual scorers, and the minority (5.0 per cent) comprised an arousal scored only by 1 manual scorer. Outside the 5402 detected arousal sequences, the automatic model detected 295 arousals that no manual scorer considered as an arousal.

**Table 4:** Electroencephalogram theta, alpha, and >16 Hz signal power during sequences where the model did and did not detect an arousal, grouped based on the number of manual scorers contributing to the sequence formation

Sleep stage	Arousal scored by		Group size (*n*)	Median (IQR) EEG frequency band power		
	Automatic model	Manual scorers (*n*)		Theta [μV^2^]	Alpha [μV^2^]	>16 Hz [μV^2^]
W	*yes*	*0–4*	347	44.2 (15.9–133.3) ^†*^	41.1 (14.7–109.6) ^†*^	124.1 (24.8–422.9) ^†*^
		*5–10*	492	38.7 (14.9–97.7) ^‡*^	32.8 (12.6–78.6) ^‡*^	74.7 (18.2–241.4) ^‡*^
	*no*	*1–4*	1489	14.1 (5.4–36.4) ^†#^	12.0 (4.4–31.3) ^†^	12.2 (4.5–35.7) ^†#^
		*5–10*	85	24.0 (8.3–66.8) ^‡#^	17.2 (6.8–42.1) ^‡^	27.5 (8.5–98.6) ^‡#^
N1–N3	*yes*	*0–4*	990	7.7 (3.5–16.7) ^*^	14.6 (5.8–36.6) ^†*^	23.0 (9.2–57.9) ^*^
		*5–10*	2688	32.5 (13.1–79.4) ^*^	22.0 (8.7–53.8) ^‡*^	22.7 (7.2–98.1) ^‡*^
	*no*	*1–4*	5660	21.7 (8.1–55.9) ^#^	11.8 (4.2–31.1) ^†#^	6.9 (2.8–15.7) ^#^
		*5–10*	823	28.2 (10.6–74.5) ^#^	17.6 (6.7–44.7) ^‡#^	16.0 (6.1–43.4) ^‡#^
R	*yes*	*0–4*	324	15.1 (6.4–35.2) ^†*^	11.6 (4.5–29.0) ^†*^	7.5 (3.5–16.0) ^†*^
		*5–10*	856	21.7 (9.1–51.7) ^*^	17.3 (6.7–42.8) ^*^	25.9 (7.7–94.5) ^‡*^
	*no*	*1–4*	1677	13.2 (5.4–29.3) ^†#^	7.7 (3.0–17.4) ^†#^	6.1 (2.5–13.7) ^†#^
		*5–10*	168	19.3 (8.2–45.3) ^#^	15.7 (5.9–37.3) ^#^	17.2 (5.9–51.8) ^‡#^

IQR = interquartile range; N1–N3 = combined non-rapid eye movement sleep stages N1, N2, and N3; R = rapid eye movement sleep stage; theta = 4–7.99 Hz; alpha = 8–13 Hz; >16 Hz = 16–40 Hz; W = wakefulness stage.

^*, †, ‡^, and ^#^ markings denote statistically significant differences (*p* < .05) between two groups with equivalent markings within the same frequency band and sleep stage. Statistical significances were evaluated with the Wilcoxon signed-rank test as the median *p*-value of 5000 random pair permutations between the compared groups.

### E‌EG activity in manually and automatically scored arousals

Across all sleep stages (W, N1–N3, and R), theta (4–7.99 Hz), alpha (8–13 Hz), and >16 Hz (16–40 Hz) EEG band powers were significantly higher during model-detected than non-detected arousals (*p* < .05; Figure 7a, c, and  e). Stratification by manual scorer agreement did not affect these findings in stage W and did not change their direction in any stage, but it modulated their statistical significances in stages N1–N3 and R (Figure 7b, d, and  f; and Table 4, *p* < .05 indicated with ^†^ and ^‡^).

**Figure 7:**
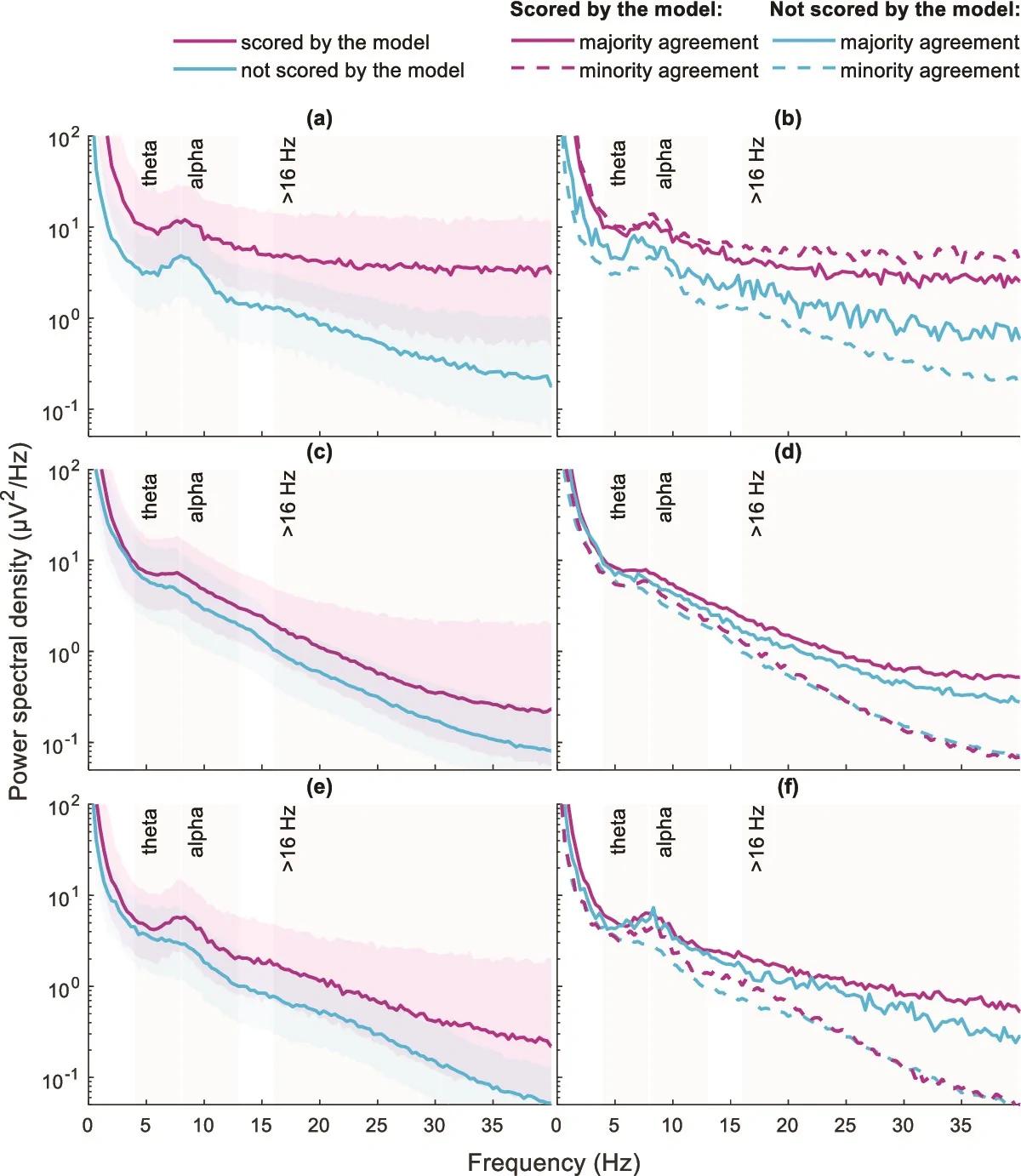
Median electroencephalogram power spectral densities (PSDs) during sequences with and without model-detected arousals in stages W (a), N1–N3 (c), and R (e). The interquartile ranges are shaded with the corresponding colors to the median curves. On the right, the PSD medians have been further computed based on whether the sequences comprised arousals from the majority (≥5) or the minority (≤4) of manual scorers in stages W (b), N1–N3 (d), and R (f).

In stages N1–N3 and R, all arousals by the manual scorer majority, regardless of model-detection, exhibited significantly higher (*p* < .05) theta, alpha, and >16 Hz EEG power than those scored by the minority (Table 4, *p* < .05 indicated with ^*^ and ^#^). In stage W, however, model-detected arousals with minority agreement showed the highest EEG power of all other groups in all frequency bands.

## Discussion

In the present study, a deep learning model was developed for the automatic scoring of arousals from sleep using EEG, chin EMG, and nasal pressure signals. The model generalized exceedingly well to an independent PSG cohort, reaching an event-by-event F1-score of 0.74 against arousals scored by the majority (≥5) of ten manual scorers. Exceeding the hypothesis, the model’s agreement with the manual scorers was overall higher than the agreement of the manual scorers with each other. This was observed both on an event-by-event basis (median F1-score of 0.61 vs 0.57, respectively) as well as in more detailed 1-s resolution (median *κ* of 0.50 vs 0.46, respectively). Moreover, as hypothesized, the EEG alpha, theta, and >16 Hz signal powers were higher during arousals scored by the model than those the model did not consider as arousals, implying the model’s learned compliance with the AASM scoring rules, where an increase in these frequencies is required to score an arousal [2].

When comparing our arousal scoring model’s performance in the MESA test set to previously published state-of-the-art models tested in non-independent datasets (i.e. hold-out test sets from the same PSG cohorts as their respective training sets), our model performed similarly or slightly better than the previous ones. In the MESA test set, our model reached event-by-event recall, precision, and F1-score values of 0.82, 0.72, and 0.77, respectively, whereas Brink-Kjaer et al. [9] and Zahid et al. [11] previously published respective values of 0.67–0.81, 0.72–0.76, and 0.70–0.76. Out of these, the higher F1-score (0.76), reported by Brink-Kjaer et al. [9], was computed using different evaluation criteria compared to ours, and therefore direct comparison is not possible. Specifically, Brink-Kjaer et al. [9] combined arousals with ≤10 s of sleep between them, whereas we removed arousals if they were not preceded by 10 s of sleep, slightly decreasing the likelihood of automatic and manual events overlapping in our case compared to Brink-Kjaer et al. [9]. The more detailed 1-s recall, precision, and F1-score values of our model in the MESA test set (0.73, 0.65, and 0.69, respectively) were also similar to, or slightly higher than those of Brink-Kjaer et al. [9] (0.73, 0.64, and 0.68, respectively).

Another state-of-the-art model by Ehrlich et al. [12], similarly trained in the MESA dataset, reached a high event-by-event F1-score of 0.81 in its MESA test set, exceeding our corresponding F1-score. However, their event-by-event F1-score evaluation differed from our study. First, they extended the manually scored arousals by 2 s before onset and 10 s after offset, increasing the probability of temporal overlap between manual and automatic labels. Second, as the automatically detected arousals were merged using a 3-s patience window, i.e. combined if there was ≤3 s of sleep between them, this further increased their likelihood of overlap (compared to our study, where arousals were removed if they were not preceded by 10 s of stable sleep). Third, they discarded model-detected arousals shorter than 15 s. Therefore, direct comparison with our results is not possible due to these differences in evaluation criteria.

In the independent SR dataset, the present model performed better than the previously published ones when independent cohorts were utilized for testing. The model of Brink-Kjaer et al. [9] has later been tested in a new cohort of 9 PSGs, reaching an event-by-event F1-score of 0.57 [10], whereas in the multi-scorer SR dataset of this study, the present model reached a median event-by-event F1-score of 0.61, ranging between 0.33–0.73 against individual scorers. The model of Ehrlich et al. [12] slightly exceeded our values, reaching event-by-event F1-scores between 0.60 and 0.76 in different independent datasets. However, it should again be noted that due to the abovementioned label adjustments, these F1-scores are not directly comparable. Similarly, Finnsson et al. [14] reported a 30-s F1-score of 0.64 in an independent cohort of 1299 PSGs [14]. However, these F1-scores are not directly comparable either, as unlike the event-by-event F1-score where true positives overlap with manually scored arousals, the 30-s F1-score does not require any overlap between the predicted and manually scored arousals.

In both datasets, the recall of the present model was higher than its precision (Table 3), meaning that the automatic model detected more arousals than the manual scorers in the MESA dataset (37 224 vs 32 801), and more than the scorer majority in the multi-scorer SR dataset (5941 vs 5111). However, in a previous study in the same SR dataset [5], each individual scorer scored 2737–8679 arousals with a median of 6447. This suggests that although model’s performance is recall-leaning, it still falls within the range of manual scoring variability.

In the multi-scorer SR dataset, the ArI ICCs between the automatic model and the individual scorers ranged from no to excellent agreement (0.07–0.87) with a median of 0.66. This was higher than the median ArI ICCs among the manual scorers (0.59). In the MESA test set, the present model scored ArIs in excellent agreement with the manually determined ArIs, reaching an ICC of 0.83. Higher agreement in the MESA than in the SR dataset was expected, as the model was trained using recordings and annotations from the MESA dataset. This also parallels previous studies that have been evaluated both in initial hold-out sets and in independent cohorts [9, 10, 13].

On average, the automatically scored arousals were 2.1 s longer than the manually scored arousals, and they started 1.3 s before and ended 0.9 s after their manually scored counterparts. This suggests that the model can capture a broader neurophysiological window around the arousal, detecting more subtle EEG changes that the human eye may overlook. However, the longer median duration of the automatically than the manually scored arousals (9.0 s vs 6.9 s, respectively) may also be explained by the MESA training data, where the median arousal duration was 9.1 s. Additionally, contrasting this longer duration, the median arousal duration in the SR dataset may have been relatively low due to one scorer mostly scoring 3-s arousals (Figure 3b and [5]). Moreover, paralleling the present results, similar or longer median arousal durations have been reported in literature (8.6–14.9 s [35–37]), and previous automatically scored arousals have similarly started earlier and ended after their manually scored counterparts (–1.37 s and +3.44 s, respectively [11]).

Compared to the manual scorers in the multi-scorer SR dataset, the model showed outstanding agreement with the majority arousal scores, considering that these majority scores were derived exclusively from the manually scored arousals without incorporating the ones scored by the model. For example, the event-by-event F1-score of the model against the majority arousal events was 0.74, whereas the manual scorers themselves reached a median event-by-event F1-score of 0.73 against the same majority events. Moreover, the 1-s *κ*-value of the model against the manual majority seconds was 0.60, whereas in the previous study in the same dataset [5], the scorers reached a median 1-s *κ*-value of 0.59 against the majority arousal seconds. In the present study, most (70.8 per cent) of the arousals detected by the model were also scored by the majority (≥5) of the manual scorers, and correspondingly, most (89.1 per cent) of the arousals that the model did not detect were only scored by the minority (≤4) of the manual scorers. These aspects indicate the model’s ability to stay consistent in its scoring, demonstrating reasonable agreement with the scorers overall.

When analyzing the model’s performance as a function of scorer agreement (Figure 5), event-by-event F1-scores of both the model and the manual scorers peaked at an agreement level of 4 scorers. This was expected, since this was also the point where the true and predicted event counts were the most similar. At higher agreement levels, the F1-scores decreased due to the progressively smaller number of true events. In contrast, the model confidence, which is not affected by the event counts, increased monotonically with scorer agreement. This indicates that the model’s internal confidence aligns with the confidence of the manual scorers, enabling uncertainty analysis and quality control of the model-detected arousals.

The EEG alpha, theta, and >16 Hz signal powers were significantly higher during arousals scored by the model compared to those not scored by the model in all stages W, N1–N3, and R (Figure 7a, c, and  e), as well as during those scored in agreement by the majority compared to the minority of the manual scorers in stages N1–N3 and R (Figure 7d and f, and Table 4). The AASM guidelines require an increase in alpha, theta, and/or >16 Hz EEG frequencies to score an arousal [2], and a larger change in these frequencies may be more easily noticed both by the model and the manual scorers. However, in stage W, model-detected arousals with majority agreement showed lower power in all EEG bands than those with minority agreement (Figure 7b and Table 4). This suggests that the model remained internally consistent by detecting arousals with increased EEG power; but among manual scorers, stronger frequency changes in stage W may increase ambiguity rather than agreement, since stage W itself is characterized by an activated EEG background [2]. Therefore, in stage W, clear contextuality is likely more important in increasing scoring agreement than strong spectral activations.

We found that the arousal-related power increases in alpha, theta, and >16 Hz bands were also individually driven by sleep stage, and that both the manual scorers and the model distinguished these stage-specific characteristics. In stage R, all model-detected arousals, and all majority arousals regardless of model-detection, were associated with >16 Hz EEG power increases, likely due to distinguishable sympathetic activations associated with stage R [38]. Out of all model-detected arousals in N1–N3, exclusively those with high agreement exhibited significant >16 Hz activation (*p* < .05), strengthening the role of high-frequency activity being an easily distinguishable arousal marker, but also emphasizing the diversity of arousal-related EEG characteristics in stages N1–N3. For example, in N1–N3, theta activity was not a significant detection marker either in high- or low-agreement arousals, whereas alpha activity was significantly (*p <* .05) increased in all detected arousals. This aligns with alpha intrusion being a known cue for arousals in non-rapid eye movement sleep [39]. Out of all model-detected arousals in stage R, however, only those with low manual agreement were associated with significantly (*p* < .05) increased theta and alpha activity. As theta and alpha rhythms are intrinsic to stage R [2], this suggests that these arousals may not be as easily distinguishable from the baseline activity.

Moreover, complying with the previously studied manual scoring agreement [4, 5], the model’s arousal scoring accuracy increased from N1 to N3. Contrasting stable sleep, where EEG high-frequency activity decreases when the sleep deepens from N1 to N3, the high-frequency EEG content of arousals increases from N1 to N3 [40]. This larger arousal-related EEG frequency shift in N3 compared to N1 may be more easily distinguishable, and thus be a reason why both the manual scoring agreement and the model performance increase correspondingly [4, 5]. Furthermore, as the arousal-related high-frequency EEG activity is already elevated during wakefulness [38], these arousals may be difficult to distinguish from the background EEG activity, which may explain why both the manual scorers and the automatic model performed the poorest when the arousals occurred in wake. In the case of the model, trained with MESA data, the poor performance in wake may also result from a specific AASM guideline amendment made after the manual scoring of the MESA dataset, regarding arousal scoring in wake (clarifying that arousals may be scored in wake epochs if they immediately precede a wake transition) [19, 21].

The present study is not without limitations, suggesting avenues for further research. Firstly, the performance of the model against the manual scorers, and the performance of the manual scorers against each other, cannot be directly compared due to different group sizes. While the performance of the model was evaluated against all ten manual scorers, the performance of each manual scorer was evaluated only against the remaining nine scorers. The smaller group size may overstate the effect of possible outliers, as they comprise a greater proportion of the group. Secondly, limb movement signals were not included in the present model, as limb movements in the MESA dataset were recorded with a combined left/right leg piezo sensor channel instead of two separate EMGs, limiting their generalizability. As arousals are often associated with PLMs [2], incorporating limb movement signals may increase the detection accuracy of these arousals. Thirdly, due to limited computational power, the scoring resolution of the model was restricted to 1-s segments. Using a shorter segment length would enable the model to place the onsets and offsets of the arousals more precisely, providing a more continuous model of sleep fragmentation.

Furthermore, although manually scored arousals were included as annotated, model-detected arousals that did not fulfill the current AASM scoring rules, specifically those lasting ˂3 s or preceded by fewer than 10 s of stable sleep, were excluded from these analyses. Their future inclusion could yield valuable insights into the neurophysiological dynamics and microstructural aspects of both sleep regulation and fragmentation. Future work could also extend our EEG spectral analysis by integrating baseline EEG comparisons to better characterize how the automatic model and different levels of manual scorer agreement respond to arousal-related changes in the underlying background EEG activity. This could provide deeper insight into the scoring decisions of both the automatic model and the manual scorers.

Moreover, although we included ArI-based analyses in our evaluations, ArI was not the primary focus of this study, as it depends not only on arousal detection but also on the TST, which itself is subject to interscorer variability. Furthermore, given that the model has not yet been validated in clinical populations, its generalizability to diverse patient characteristics, pathologies, altered sleep architecture, or atypical EEG patterns remains to be established. Therefore, validation in clinical cohorts and outcomes, using clinically relevant metrics, such as the ArI, will be essential in future research. Such analyses would help determine whether the model provides reliable estimates not only of event-level arousal agreement, but also of clinically interpretable summary measures that may be used in patient assessment and disease characterization. Furthermore, as the present model was exclusively trained on adult data, pediatric arousals were not considered in its validation either. Given the differences in scoring criteria and underlying sleep physiology, extending the approach to pediatric populations will require a dedicated, population-specific model, and evaluation.

Altogether, the model’s compliance with expert scorers, alignment with AASM guidelines [2], and sensitivity to physiologically relevant EEG features highlight its clinical potential for identifying arousals to assess sleep fragmentation from PSGs, which is a central aspect of diagnosis in disorders such as sleep apnea, insomnia, and movement disorders [2]. In practical settings, the automation of arousal identification can reduce the time and resource burden of manual scoring and enable large-scale or longitudinal analyses that are currently difficult to perform.

## Conclusion

The presented automatic arousal scoring model was shown to reflect genuine arousal scoring practices, and it generalized exceedingly well to independent PSG data scored by multiple expert scorers. The model achieved an event-by-event F1-score of 0.74 against arousals scored by the majority of ten scorers, on par with the scorers themselves (0.73). Moreover, the EEG alpha, theta, and >16 Hz spectral powers were greater during segments the model considered as arousals than during those it did not, indicating meaningful physiological distinctions and compliance with the AASM arousal scoring rules, where an increase in these frequencies is required.

As the model’s performance is on par with manual scorers in an independent dataset, this model can reliably be used to score arousals from different sleep recordings in research environments.

## Data Availability

The source codes of the arousal detection model presented in this study are publicly available at https://github.com/UEF-SmartSleepLab/slf-arousalscoring and the MESA dataset used in the model training is publicly available at https://sleepdata.org/datasets/mesa [18].
